# A Dictionary of Terms Used in Medicine and the Collateral Sciences

**Published:** 1845-11

**Authors:** 


*A Dictionary of Terms used in Medicine and the Collateral Sciences.* By Richard Hoblyn, A. M., Oxon. First American from the second London edition, with numerous additions, by Isaac Hays, M. D., editor of the American Journal of the Medical Sciences. Philadelphia: Lea & Blanchard, 1845. (From the publishers.)

This is a neat duodecimo volume, intended as a convenient manual of reference, for the definition of medical terms and their etymology. It is not intended to take the place of larger works, but from its conciseness and clearness when definitions and not essays, are required, is more convenient for constant reference. To the English edition the American editor has made additions and alterations, adapting it to the wants of the American readers. He has introduced the native medicinal plants, and brought the work into conformity with the U. S. Pharmacopœe. This little volume should be on the table of every practitioner, and in the possession of every medical student. (For sale by Brautigam & Keen, Lake street, Chicago.) Ed.

